# Supplementary material for: Effects of iron supplementation on cognitive development in school-age children: Systematic review and meta-analysis
Source: PLoS One. 2023 Jun 27;18(6):e0287703. doi: 10.1371/journal.pone.0287703 (PMC10298800; doi:10.1371/journal.pone.0287703)
Supplement: S1 Table — (DOCX) [file pone.0287703.s002.docx]

**S1 Table.** Medline search strategy for the effects of iron supplementation on cognitive development in school-age children

| **NAME OF DATABASE (interface):** MEDLINE (via the PubMed interface) | | |
| --- | --- | --- |
| **Concept** | **Line number** | **Search strategy** |
| Concept 1: Cognition | Cognition | "cognition"[MeSH Terms] OR "cognition*"[TW] OR "cognitive*"[TW] |
|  | Cognitive neuroscience | "cognitive neuroscience"[MeSH Terms] |
|  | Child development | "child development"[MeSH Terms] OR "child develop*"[TW] |
|  | Language development | "language development"[MeSH Terms] OR (language [TW] AND develop*[TW]) OR (language[TW] AND learn*[TW]) OR (language[TW] AND train*[TW]) OR (language[TW] AND acquisition[TW]) |
|  | Intelligence tests | "intelligence tests"[MeSH Terms] OR (intelligence[TW] AND test*[TW]) OR (intelligence[TW] AND measurement*[TW]) |
|  | Intelligence quotient | ("intelligence"[MeSH Terms] OR intelligen*[TW]) AND (quotient*[TW]) |
|  | Neuropsychological test | "neuropsychological tests"[MeSH Terms] OR (neuropsychological[TW] AND test*[TW]) OR (neuropsychologic[TW] AND assessment*[TW]) OR (neuropsychologic[TW] AND examination*[TW]) |
|  | Wechsler scales | "wechsler scales"[MeSH Terms] OR (wechsler[TW] AND scale*[TW]) OR "wms iv nl"[TW] OR "wisc v"[TW] OR "wisc iv"[TW] OR "wais r"[TW] OR "wechsler preschool and primary scale of intelligence"[TW] OR wppsi[TW] |
|  | Stanford Binet test | "stanford binet test"[MeSH Terms] OR (test*[TW] AND binet[TW]) |
|  | Developmental psychology | "psychology, developmental"[MeSH Terms] OR (developmental [TW] AND psychology [TW]) |
|  | Academic achievement/ success | "academic success"[MeSH Terms] OR (academic [TW] AND success*[TW]) OR "achievement"[MeSH Terms] OR (academic [TW] AND achievement*[TW]) OR (education* [TW] AND success* [TW]) OR (education* [TW] AND achievement* [TW) |
|  | Academic performance | "academic performance"[MeSH Terms] OR (academic [TW] AND performance*[TW]) OR (academic[TW] AND test*[TW] AND score*[TW]) OR (educational[TW] AND performance*[TW]) OR (educational[TW] AND test*[TW] AND score*[TW]) OR (educational[TW] AND test*[TW] AND performance*[TW]) |
|  | Learning curve | "learning curve"[MeSH Terms] OR "learning curve"[TW] |
|  | Psychomotor performance | "psychomotor performance"[MeSH Terms] OR (psychomotor[TW] AND performance*[TW]) OR (visual[TW] AND motor[TW] AND performance*[TW]) OR (visuomotor[TW] AND coordination[TW]) OR (perceptual[TW] AND motor[TW] AND performance[TW]) |
|  | Aptitude tests | "aptitude tests"[MeSH Terms] OR ("aptitude"[TW] AND "test*"[TW]) |
|  | Multitasking behavior | "multitasking behavior"[MeSH Terms] OR ("multitask*"[TW] AND "behavior*"[TW]) |
|  | Underachievement | "underachievement"[MeSH Terms] OR "underachiev*"[TW] |
|  | Executive function | "executive function"[MeSH Terms] OR (executive[TW] AND function*[TW]) OR (executive[TW] AND control*[TW]) |
|  | LEARNING | "learning"[MeSH Terms] OR "learning"[TW] OR "learn"[TW] OR "learnings"[TW] OR "learns"[TW] OR "verbal learning"[MeSH Terms] OR "serial learning"[MeSH Terms] OR "memory and learning tests"[MeSH Terms] |
|  | PROBLEM SOLVING | "problem solving"[MeSH Terms] OR (problem[TW] AND solving [TW]) |
|  | THINKING | "thinking"[MeSH Terms] OR "thinking"[TW] |
| Concept 2: Schoolchild | Child | "child"[MeSH Terms] OR "child"[TW] OR "children"[TW] OR "childrens"[TW] OR "childs"[TW] OR boy[tw] OR boys[tw] OR boyhood[tw] OR girl[tw] OR girls[tw] OR girlhood[tw] OR teen[tw] OR teens[tw] OR teenager*[tw] OR (pre-adolescen*[TW]) OR (preadolescen*[TW]) OR "preteen*"[TW] |
|  | School/School-child | "schools"[MeSH Terms] OR "school*"[TW] OR "education"[TW] OR "Elementary"[TW] |
|  | Student | "students"[MeSH Terms] OR "student*"[TW] |
|  | Pupil | "pupil"[MeSH Terms] OR "pupil"[TW] OR "pupils"[TW] |
| Concept 3: Iron Supplementation | Iron supplementation | "iron"[MeSH Terms] OR "iron"[TW] OR "iron, dietary"[MeSH Terms] OR "ferric compounds"[MeSH Terms] OR "ferric*"[TW] OR "ferrous compounds"[MeSH Terms] OR "ferrous*"[TW] OR "fe"[TW] |
|  | Anemia and Iron deficiency | "anemia"[MeSH Terms] OR "anemia*"[TW] OR "anaemia*"[TW] OR "anemia, iron deficiency"[MeSH Terms] OR "iron-deficiency"[TW] OR "iron binding proteins"[MeSH Terms] |
|  | Dietary Supplements | "dietary supplements"[MeSH Terms] OR ("dietary"[TW] AND "supplement*"[TW]) |
|  | Diet supplementation | ("diet"[MeSH Terms] OR "diet"[TW]) AND ("supplement*"[TW]) |
|  | Mineral supplementation | ("minerals"[MeSH Terms] OR "minerals"[TW] OR "mineral"[TW]) AND "supplement*"[TW] |
|  | Multi-nutrient supplement | ("multinutrient"[TW] OR "multinutrients"[TW]) AND "supplement*"[TW] |
|  | Micronutrient supplementation | ("micronutrients"[MeSH Terms] OR "micronutrient*"[TW] OR "micronutriments"[TW] OR "trace elements"[MeSH Terms] OR ("trace"[TW] AND "element*"[TW])) AND ("supplement*"[TW]) |
| Filters applied |  | Clinical Trial |
|  |  | Randomized Controlled Trial |
|  |  | Child: birth-18 years |
|  |  | Child: 6-12 years |
