# Supplementary material for: Effects of iron supplementation on cognitive development in school-age children: Systematic review and meta-analysis
Source: PLoS One. 2023 Jun 27;18(6):e0287703. doi: 10.1371/journal.pone.0287703 (PMC10298800; doi:10.1371/journal.pone.0287703)
Supplement: S2 Table — (DOCX) [file pone.0287703.s003.docx]

**S2 Table.** Embase search strategy for the effects of iron supplementation on cognitive development in school-age children

| **NAME OF DATABASE (interface):** Embase (via the embase.com) | | |
| --- | --- | --- |
| **Concept** | **Line number** | **Search strategy** |
| Concept 1: Cognition | Cognition | 'cognition'/exp OR 'cognitive function test'/exp OR 'cognition assessment'/exp OR cognition*:ti,ab,kw OR cognitive*:ti,ab,kw |
|  | Cognitive development | 'cognitive development'/exp |
|  | Cognitive neuroscience | 'cognitive neuroscience'/exp |
|  | Child development | 'child development'/exp OR 'child develop*':ti,ab,kw |
|  | Language development | 'language development'/exp OR (('language'/exp OR 'language':ti,ab,kw) AND ('development'/exp OR 'develop*':ti,ab,kw)) OR (('language'/exp OR 'language':ti,ab,kw) AND ('learning'/exp OR 'learn*':ti,ab,kw)) OR (('language'/exp OR 'language':ti,ab,kw) AND ('training'/exp OR 'train*':ti,ab,kw)) OR (('language'/exp OR 'language':ti,ab,kw) AND ('acquisition'/exp OR 'acquisition':ti,ab,kw)) |
|  | Intelligence tests | 'intelligence test'/exp OR (('intelligence'/exp OR intelligence:ti,ab,kw) AND ('test'/exp OR test*:ti,ab,kw)) OR (('intelligence'/exp OR 'intelligence':ti,ab,kw) AND ('measurement'/exp OR 'measurement*':ti,ab,kw)) |
|  | Intelligence quotient | 'intelligence quotient'/exp OR (('intelligence'/exp OR intelligen*:ti,ab,kw) AND quotient:ti,ab,kw) OR iq:ti,ab,kw |
|  | Neuropsychological test | 'neuropsychological test'/exp OR (neuropsychological:ti,ab,kw AND ('test'/exp OR test*:ti,ab,kw)) OR (neuropsychological:ti,ab,kw AND ('assessment'/exp OR assessment*:ti,ab,kw)) OR (neuropsychological:ti,ab,kw AND ('examination'/exp OR examination*:ti,ab,kw)) |
|  | Wechsler scales | 'wechsler intelligence scale'/exp OR 'wechsler intelligence scale for children'/exp OR (wechsler:ti,ab,kw AND ('scale'/exp OR scale*:ti,ab,kw)) OR wisc:ti,ab,kw OR 'wms iv nl':ti,ab,kw OR 'wisc v':ti,ab,kw OR 'wais r':ti,ab,kw OR wppsi:ti,ab,kw |
|  | Stanford Binet test | 'stanford-binet intelligence scale'/exp OR (stanford:ti,ab,kw AND binet:ti,ab,kw AND ('scale'/exp OR scale*:ti,ab,kw)) OR (binet:ti,ab,kw AND ('test'/exp OR test*:ti,ab,kw)) |
|  | Developmental psychology | 'developmental psychology'/exp OR (developmental:ti,ab,kw AND ('psychology'/exp OR psycholog*:ti,ab,kw)) |
|  | Academic achievement/ success | 'academic achievement'/exp OR (academic:ti,ab,kw AND ('achievement'/exp OR achievement*:ti,ab,kw)) OR 'academic success'/exp OR (academic:ti,ab,kw AND ('success'/exp OR success*:ti,ab,kw)) OR (education*:ti,ab,kw AND ('success'/exp OR success*:ti,ab,kw)) OR (('education'/exp OR education*:ti,ab,kw) AND ('achievement'/exp OR achievement*:ti,ab,kw)) |
|  | Academic performance | academic:ti,ab,kw AND ('performance'/exp OR performance*:ti,ab,kw) OR (academic:ti,ab,kw AND ('test'/exp OR test*:ti,ab,kw) AND ('score'/exp OR score:ti,ab,kw)) OR (education*:ti,ab,kw AND ('performance'/exp OR performance*:ti,ab,kw)) OR (education*:ti,ab,kw AND ('test'/exp OR test*:ti,ab,kw) AND ('score'/exp OR score*:ti,ab,kw)) |
|  | Learning curve | 'learning curve'/exp OR 'learning curve':ti,ab,kw |
|  | Psychomotor performance | 'psychomotor performance'/exp OR (psychomotor:ti,ab,kw AND ('performance'/exp OR performance:ti,ab,kw)) OR 'visuomotor coordination'/exp OR (visuomotor:ti,ab,kw AND ('coordination'/exp OR coordination:ti,ab,kw)) OR 'perceptual motor performance' OR (perceptual:ti,ab,kw AND ('motor'/exp OR motor:ti,ab,kw) AND ('performance'/exp OR performance*:ti,ab,kw)) |
|  | Aptitude tests | 'aptitude test'/exp OR (('aptitude'/exp OR aptitude:ti,ab,kw) AND ('test'/exp OR test*:ti,ab,kw)) |
|  | Multitasking behavior | ('multitasking'/exp OR 'multitask*':ti,ab,kw) AND ('behavior'/exp OR behavior*:ti,ab,kw) |
|  | Underachievement | 'academic underachievement'/exp OR underachievement:ti,ab,kw |
|  | Executive function | 'executive function'/exp OR 'executive function test'/exp OR (('executive'/exp OR executive:ti,ab,kw) AND ('function'/exp OR function*:ti,ab,kw)) OR (('executive'/exp OR executive:ti,ab,kw) AND ('control'/exp OR control:ti,ab,kw)) |
|  | LARNING | 'learning'/exp OR 'learning':ti,ab,kw OR 'learn':ti,ab,kw OR 'learnings':ti,ab,kw OR 'learns':ti,ab,kw OR 'verbal learning'/exp OR 'verbal learning':ti,ab,kw OR 'sequence learning'/exp OR 'sequence learning':ti,ab,kw OR 'memory learning tests':ti,ab,kw |
|  | PROBLEM SOLVING | 'problem solving'/exp OR (problem:ti,ab,kw AND solving:ti,ab,kw) |
|  | THINKING | 'thinking'/exp OR thinking:ti,ab,kw |
| Concept 2: Schoolchild | Child | 'child'/exp OR child*:ti,ab,kw OR 'boy'/exp OR boy*:ti,ab,kw OR 'girl'/exp OR girl*:ti,ab,kw OR 'preadolescence'/exp OR preadolescence:ti,ab,kw OR 'pre adolescence':ti,ab,kw |
|  | School/School-child | 'school'/exp OR school*:ti,ab,kw OR 'education'/exp OR education*:ti,ab,kw OR elementary:ti,ab,kw OR 'elementary student'/exp OR 'school child'/exp |
|  | Student | 'student'/exp OR student*:ti,ab,kw |
|  | Pupil | pupil:ti,ab,kw OR pupils:ti,ab,kw |
| Concept 3: Iron Supplementation | Iron supplementation | 'iron'/exp OR iron:ti,ab,kw OR 'iron supplementation'/exp OR 'iron intake'/exp OR 'ferric ion'/exp OR ferric*:ti,ab,kw OR 'ferrous ion'/exp OR ferrous*:ti,ab,kw OR fe:ti,ab,kw |
|  | Anemia and Iron deficiency | 'anemia'/exp OR anemia:ti,ab,kw OR 'iron deficiency'/exp OR 'iron deficiency anemia'/exp OR 'iron binding protein'/exp OR anaemia:ti,ab,kw OR hypochromic:ti,ab,kw |
|  | Iron therapy | 'iron therapy'/exp |
|  | Dietary Supplements | 'dietary supplement'/exp OR (dietary:ti,ab,kw AND supplement*:ti,ab,kw) |
|  | Diet supplementation | 'diet supplementation'/exp OR (('diet'/exp OR diet:ti,ab,kw) AND ('supplementation'/exp OR 'supplement*':ti,ab,kw)) |
|  | Mineral supplementation | 'mineral supplementation'/exp AND ('mineral'/exp OR mineral:ti,ab,kw) AND ('supplementation'/exp OR supplement*:ti,ab,kw) |
|  | Multi-nutrient supplement | 'nutrition supplement'/exp OR (('nutrition'/exp OR nutrition:ti,ab,kw) AND supplement*:ti,ab,kw) OR (multinutrient:ti,ab,kw AND supplement*:ti,ab,kw) |
|  | Micronutrient supplementation | ('micronutrient'/exp OR micronutrient:ti,ab,kw) AND ('supplementation'/exp OR 'supple-ment*':ti,ab,kw) OR micronutriments:ti,ab,kw OR 'trace element'/exp OR (('trace'/exp OR trace:ti,ab,kw) AND ('element'/exp OR element*:ti,ab,kw)) OR 'mineral deficiency'/exp OR (('mineral' OR mineral:ti,ab,kw) AND ('deficiency'/exp OR 'deficiency':ti,ab,kw)) |
| Filters applied |  | Controlled clinical trial OR Randomized controlled trial  AND  Child OR School |
