# Supplementary material for: Effects of iron supplementation on cognitive development in school-age children: Systematic review and meta-analysis
Source: PLoS One. 2023 Jun 27;18(6):e0287703. doi: 10.1371/journal.pone.0287703 (PMC10298800; doi:10.1371/journal.pone.0287703)
Supplement: S3 Table — (DOCX) [file pone.0287703.s004.docx]

**S3 Table.** Scopus search strategy for the effects of iron supplementation on cognitive development in school-age children

| **NAME OF DATABASE (interface):** Scopus (via the Scopus.com) | | |
| --- | --- | --- |
| **Concept** | **Line number** | **Search strategy** |
| Concept 1: Cognition | Cognition | TITLE-ABS-KEY (cognition* OR cognitive* ) |
|  | Child development | TITLE-ABS-KEY ("child develop*") |
|  | Language development | TITLE-ABS-KEY ( ( language AND develop* ) OR ( language AND learn* ) OR ( language AND train* ) OR ( language AND acquisition )) |
|  | Intelligence tests | TITLE-ABS-KEY ( ( intelligence AND test* ) OR ( intelligence AND measurement* ) ) |
|  | Intelligence quotient | TITLE-ABS-KEY ( intelligen* AND quotient* ) |
|  | Neuropsychological test | TITLE-ABS-KEY ( ( (neuropsychological AND test* ) OR ( neuropsychological AND assessment* ) OR (neuropsychological AND examination ) ) |
|  | Wechsler scales | TITLE-ABS-KEY ( ( wechsler AND scale* ) OR "wms iv nl" OR "wisc v" OR "wisc iv" OR "wais r" OR "wechsler preschool and primary scale of intelligence" OR "wppsi" ) |
|  | Stanford Binet test | TITLE-ABS-KEY ( ( binet AND test* ) OR ( stanford AND binet ) ) |
|  | Developmental psychology | TITLE-ABS-KEY ( Developmental AND psychology ) |
|  | Academic achievement/ success | TITLE-ABS-KEY ( ( academic AND success* ) OR ( academic AND achievement*) OR ( education* AND success* ) OR ( education* AND achievement* ) ) |
|  | Academic performance | TITLE-ABS-KEY ( ( academic AND performance* ) OR ( academic AND test* AND score* ) OR ( education* AND performance* ) OR ( education* AND test* AND score* ) ) |
|  | Learning curve | TITLE-ABS-KEY ( "learning curve" ) |
|  | Psychomotor performance | TITLE-ABS-KEY ( ( psychomotor AND performance*) OR (visual AND motor AND performance* ) OR ( visuomotor AND coordination ) OR ( perceptual AND motor AND performance* ) ) |
|  | Aptitude tests | TITLE-ABS-KEY ( aptitude AND test* ) |
|  | Multitasking behavior | TITLE-ABS-KEY ( multitask* AND behavior* ) |
|  | Underachievement | TITLE-ABS-KEY ( underachieve* ) |
|  | Executive function | TITLE-ABS-KEY (( executive AND function* ) OR ( executive AND control* ) ) |
|  | LARNING | TITLE-ABS-KEY ( learning OR learn OR learnings OR learns OR ( training AND memory ) OR "verbal learning" OR "serial learning" OR "memory and learning tests" ) |
|  | PROBLEM SOLVING | TITLE-ABS-KEY ( problem AND solving ) |
|  | THINKING | TITLE-ABS-KEY ( thinking) |
| Concept 2: Schoolchild | Child | TITLE-ABS-KEY ( child OR children OR boy OR boys OR boyhood OR girl OR girls OR girlhood OR teen OR teens OR teenager* OR ( pre-adolescen* ) OR ( preadolescen* ) OR preteen* ) |
|  | School/School-child | TITLE-ABS-KEY ( school* OR education OR elementary ) |
|  | Student | TITLE-ABS-KEY ( student* ) |
|  | Pupil | TITLE-ABS-KEY ( pupil OR pupils ) |
| Concept 3: Iron Supplementation | Iron supplementation | TITLE-ABS-KEY ( iron OR ferric* OR ferrous* OR fe ) |
|  | Anemia and Iron deficiency | TITLE-ABS-KEY ( anemia* OR anaemia* OR "iron deficiency" OR "iron-deficiency" OR "iron binding proteins" ) |
|  | Dietary Supplements | TITLE-ABS-KEY ( dietary AND supplement* ) |
|  | Diet supplementation | TITLE-ABS-KEY ( diet AND supplement* ) |
|  | Mineral supplementation | TITLE-ABS-KEY ( minerals AND supplement* ) |
|  | Multi-nutrient supplement | TITLE-ABS-KEY ( ( multinutrient OR multinutrients ) AND supplement* ) |
|  | Micronutrient supplementation | TITLE-ABS-KEY ( ( ( micronutrient* OR micronutriments ) AND ( supplement* ) ) OR ( trace AND element* AND supplement* ) ) |
| Filters applied |  | ***( INDEXTERMS ( "clinical trial*" OR "randomized controlled trial*" OR "controlled clinical trial*" OR "random allocation" OR "Double-Blind Method" OR "Single-Blind Method" OR "Cross-Over Studies" OR "Placebo*" OR "multicenter study" OR "double blind procedure" OR "single blind procedure" OR "crossover procedure" OR "controlled study" OR "randomization" ) ) OR ( TITLE-ABS-KEY ( "clinical trial*" OR "randomized controlled trial*" OR "controlled clinical trial*" OR "random allocation" OR "randomly allocated" OR "allocated randomly" OR "Cross-Over Studies" OR "Placebo*" OR "cross-over trial*" OR "single blind" OR "double blind" OR "factorial design" OR "factorial trial*" ) ) OR ( TITLE-ABS ( clinical AND trial* OR trial* OR rct* OR random* OR blind* ) )*** |
|  |  |  |
|  |  |  |
