# Supplementary material for: Effects of iron supplementation on cognitive development in school-age children: Systematic review and meta-analysis
Source: PLoS One. 2023 Jun 27;18(6):e0287703. doi: 10.1371/journal.pone.0287703 (PMC10298800; doi:10.1371/journal.pone.0287703)
Supplement: S4 Table — (DOCX) [file pone.0287703.s005.docx]

**S4 Table.** Web of Science search strategy for the effects of iron supplementation on cognitive development in school-age children

| **NAME OF DATABASE (interface):** Web of Science (via the apps.webofknowledge.com) | | |
| --- | --- | --- |
| **Concept** | **Line number** | **Search strategy** |
| Concept 1: Cognition | Cognition | ts=cognition* OR ts =Cognitive* |
|  | Child development | ts=(Child develop*) |
|  | Language development | ts=(Language AND develop*) OR ts=(Language AND learn*) OR ts=(Language AND train*) OR ts=(Language AND acquisition) |
|  | Intelligence tests | ts=(Intelligence AND test*) OR ts=(Intelligence AND measurement*) |
|  | Intelligence quotient | ts=(Intelligence quotient) |
|  | Neuropsychological test | ts=(Neuropsychological AND test*) OR ts=(neuropsychological AND assessment*) OR ts=(neuropsychological AND examination*) |
|  | Wechsler scales | ts=(Wechsler AND scale*) OR ts=(wms iv nl) OR ts=(wisc v) OR ts=(wisc iv) OR ts=(wais r) OR ts= (wppsi) |
|  | Stanford Binet test | ts=(Binet AND test*) |
|  | Developmental psychology | ts=( developmental AND psychology) |
|  | Academic achievement/ success | ts=(academic AND achievement*) OR ts=(academic AND success*) OR ts=(education* AND success*) OR ts=(education* AND achievement*) |
|  | Academic performance | ts=(academic AND performance*) OR ts=(academic AND test* AND score*) OR ts=(educational AND performance*) OR ts=(educational AND test* AND score*) |
|  | Learning curve | ts="learning curve" |
|  | Psychomotor performance | ts=(psychomotor AND performance*) OR ts= (visual AND motor AND performance*) OR ts=(visuomotor AND coordination) OR ts= (perceptual AND motor AND performance*) |
|  | Aptitude tests | ts=(aptitude AND test*) |
|  | Multitasking behavior | ts=(multitask* AND behavior*) |
|  | Underachievement | ts=( underachieve*) |
|  | Executive function | ts=(executive function*) OR ts=(executive control*) |
|  | LARNING | ts=(learning) OR ts=(learn) OR ts=(learnings) OR ts=(learns) OR ts=("training memory") OR ts=("verbal learning") OR ts=("serial learning") OR ts=("memory learning tests") |
|  | PROBLEM SOLVING | ts=(problem AND solving) |
|  | THINKING | ts=(thinking) |
| Concept 2: Schoolchild | Child | ts=(child*) OR ts=(boy*) OR ts=(girl*) OR ts=(teen) OR ts=(teens) OR ts=(teenager*) OR ts=(pre-adolescen*) OR ts=(preadolescen*) OR ts=(preteen*) |
|  | School/School-child | ts=(school*) OR ts=(education) OR ts=(elementary) |
|  | Student | ts=(student*) |
|  | Pupil | ts=(pupil) OR ts=(pupils) |
| Concept 3: Iron Supplementation | Iron supplementation | ts=(iron) OR ts=(ferric*) OR ts=(ferrous*) OR ts=(fe) |
|  | Anemia and Iron deficiency | ts=(anemia) OR ts=(anaemia) |
|  | Dietary Supplements | ts=(dietary AND supplement*) |
|  | Diet supplementation | ts=(diet AND supplement*) |
|  | Mineral supplementation | ts=(mineral AND supplement*) |
|  | Multi-nutrient supplement | ts=(multinutrients AND supplement*) |
|  | Micronutrient supplementation | ts=(micronutrient* AND supplement*) OR ts=(micronutriments AND supplement*) OR ts= (trace AND element* AND supplement*) |
| Filters applied | Clinical Trial  Randomized Controlled Trial | ts= ((clinical near trial* or crossover or "cross over") or ((single* or doubl* or trebl* or tripl*) near (blind* or mask* or dummy)) or (singleblind* or doubleblind* or trebleblind* or tripleblind* or placebo* or random*) or (random* AND (allocat* OR assign*)) or (controlled trial*) or (follow-up stud*) or (prospective stud*)) |
