# Supplementary material for: Effects of iron supplementation on cognitive development in school-age children: Systematic review and meta-analysis
Source: PLoS One. 2023 Jun 27;18(6):e0287703. doi: 10.1371/journal.pone.0287703 (PMC10298800; doi:10.1371/journal.pone.0287703)
Supplement: S5 Table — (DOCX) [file pone.0287703.s006.docx]

**S5 Table.** Cochrane Library search strategy for the effects of iron supplementation on cognitive development in school-age children

| **NAME OF DATABASE (interface):** Cochrane Library (via www.cochranelibrary.com) | | |
| --- | --- | --- |
| **Concept** | **Line number** | **Search strategy** |
| Concept 1: Cognition | Cognition | (cognition* OR cognitive*):ti,ab,kw |
|  | Child development | ("child develop*"):ti,ab,kw |
|  | Language development | ((language AND develop*) OR (language AND learn*) OR (language AND train*) OR (language AND acquisition)):ti,ab,kw |
|  | Intelligence tests | ((intelligence AND test*) OR (intelligence AND measurement*)):ti,ab,kw |
|  | Intelligence quotient | (intelligen* AND quotient*):ti,ab,kw |
|  | Neuropsychological test | ((neuropsychological AND test*) OR (neuropsychological AND assessment*) OR (neuropsychological AND examination)):ti,ab,kw |
|  | Wechsler scales | ((wechsler AND scale*) OR "wms iv nl" OR "wisc v" OR "wisc iv" OR "wais r" OR "wechsler preschool and primary scale of intelligence" OR "wppsi"):ti,ab,kw |
|  | Stanford Binet test | ((binet AND test*) OR (stanford AND binet)):ti,ab,kw |
|  | Developmental psychology | (Developmental AND psychology):ti,ab,kw |
|  | Academic achievement/ success | ((academic AND success*) OR (academic AND achievement*) OR (education* AND success*) OR (education* AND achievement*)):ti,ab,kw |
|  | Academic performance | ((academic AND performance*) OR (academic AND test* AND score*) OR (educational AND performance*) OR (educational AND test* AND score*)):ti,ab,kw |
|  | Learning curve | "learning curve":ti,ab,kw |
|  | Psychomotor performance | ((psychomotor AND performance*) OR (visual AND motor AND performance*) OR (visuomotor AND coordination) OR (perceptual AND motor AND performance*)):ti,ab,kw |
|  | Aptitude tests | (aptitude AND test*):ti,ab,kw |
|  | Multitasking behavior | (multitask* AND behavior*):ti,ab,kw |
|  | Underachievement | (underachieve*):ti,ab,kw |
|  | Executive function | ((executive AND function*) OR (executive AND control*)):ti,ab,kw |
|  | LARNING | (learning OR learn OR learnings OR learns OR (training AND memory) OR "verbal learning" OR "serial learning" OR "memory and learning tests"):ti,ab,kw |
|  | PROBLEM SOLVING | (problem AND solving):ti,ab,kw |
|  | THINKING | (thinking):ti,ab,kw |
| Concept 2: Schoolchild | Child | (child OR children OR boy OR boys OR boyhood OR girl OR girls OR girlhood OR teen OR teens OR teenager* OR preadolescen* OR preteen*):ti,ab,kw |
|  | School/School-child | (school* OR education OR elementary):ti,ab,kw |
|  | Student | (student*):ti,ab,kw |
|  | Pupil | (pupil OR pupils):ti,ab,kw |
| Concept 3: Iron Supplementation | Iron supplementation | (iron OR ferric* OR ferrous* OR fe):ti,ab,kw |
|  | Anemia and Iron deficiency | (anemia* OR anaemia* OR "iron deficiency" OR "iron-deficiency" OR "iron binding proteins"):ti,ab,kw |
|  | Iron therapy |  |
|  | Dietary Supplements | (dietary AND supplement*):ti,ab,kw |
|  | Diet supplementation | (diet AND supplement*):ti,ab,kw |
|  | Mineral supplementation | (minerals AND supplement*):ti,ab,kw |
|  | Multi-nutrient supplement | ((multinutrient OR multinutrients) AND supplement*):ti,ab,kw |
|  | Micronutrient supplementation | (((micronutrient* OR micronutriments) AND (supplement*)) OR (trace AND element* AND supplement*)):ti,ab,kw |
| Filters applied |  | Trials |
