# Supplementary figures and images for: Effects of iron supplementation on cognitive development in school-age children: Systematic review and meta-analysis
Source: PLoS One. 2023 Jun 27;18(6):e0287703. doi: 10.1371/journal.pone.0287703 (PMC10298800; doi:10.1371/journal.pone.0287703)

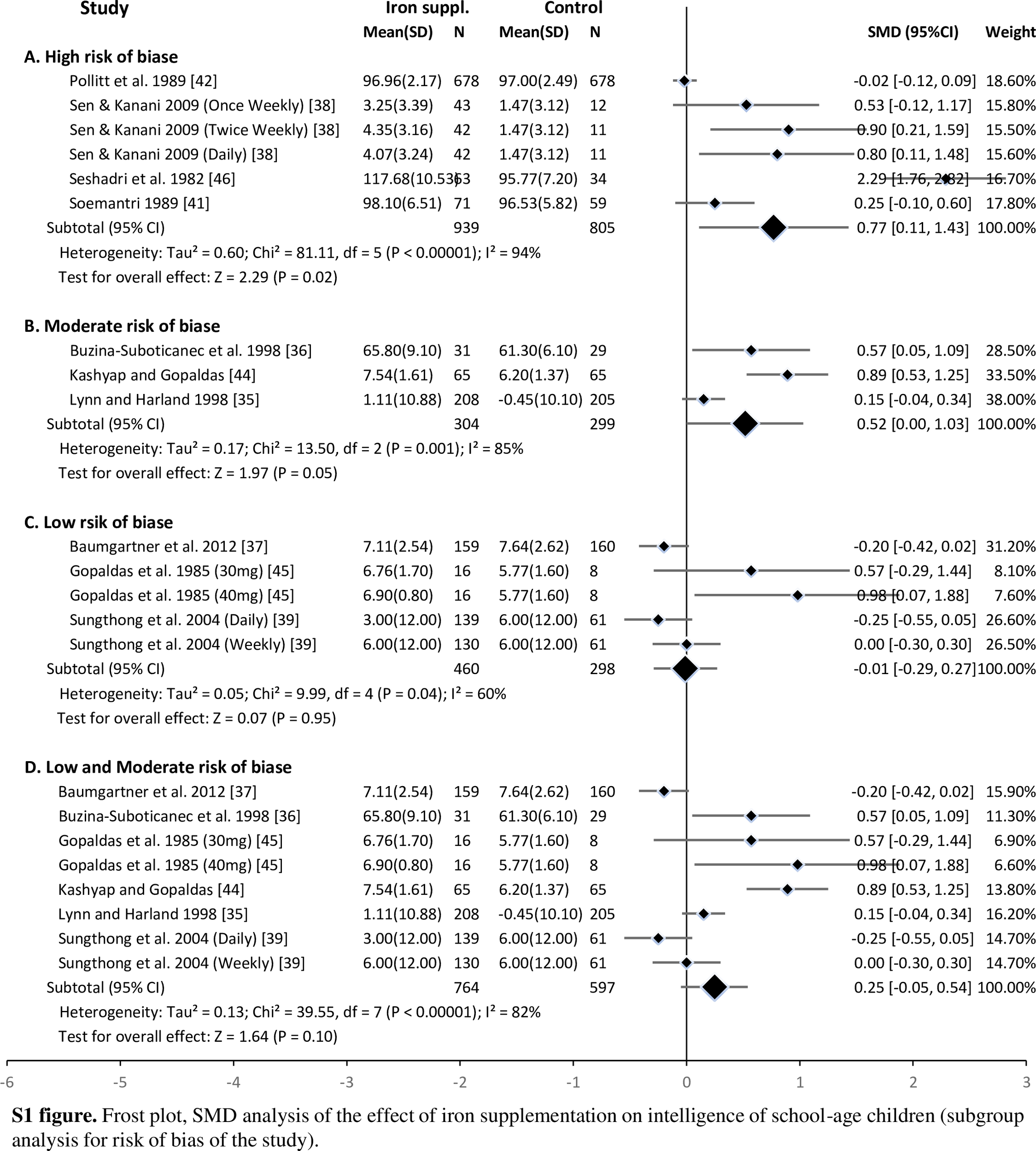

Supplement: S1 Fig — (TIF) [file pone.0287703.s007.tif]

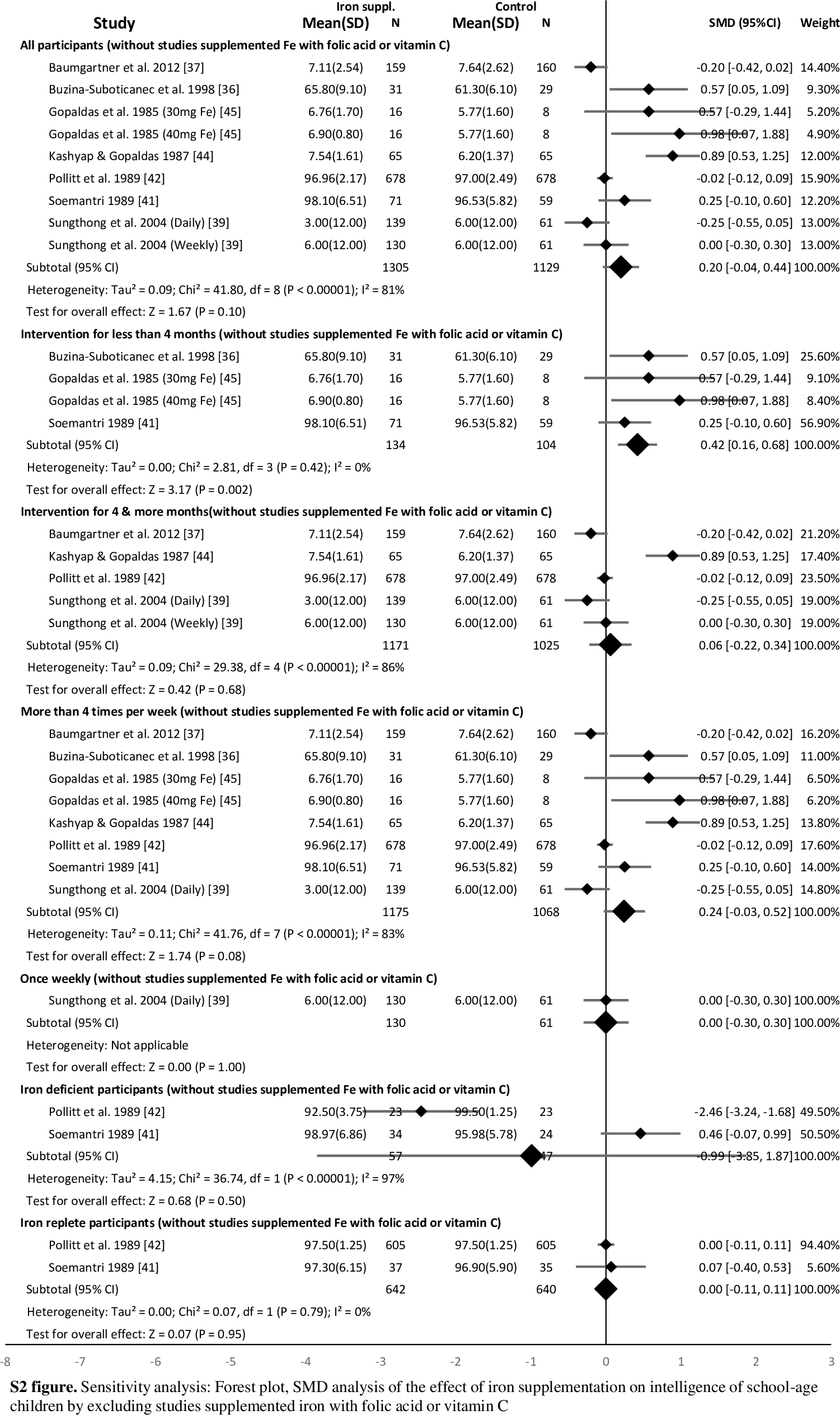

Supplement: S2 Fig — (TIF) [file pone.0287703.s008.tif]

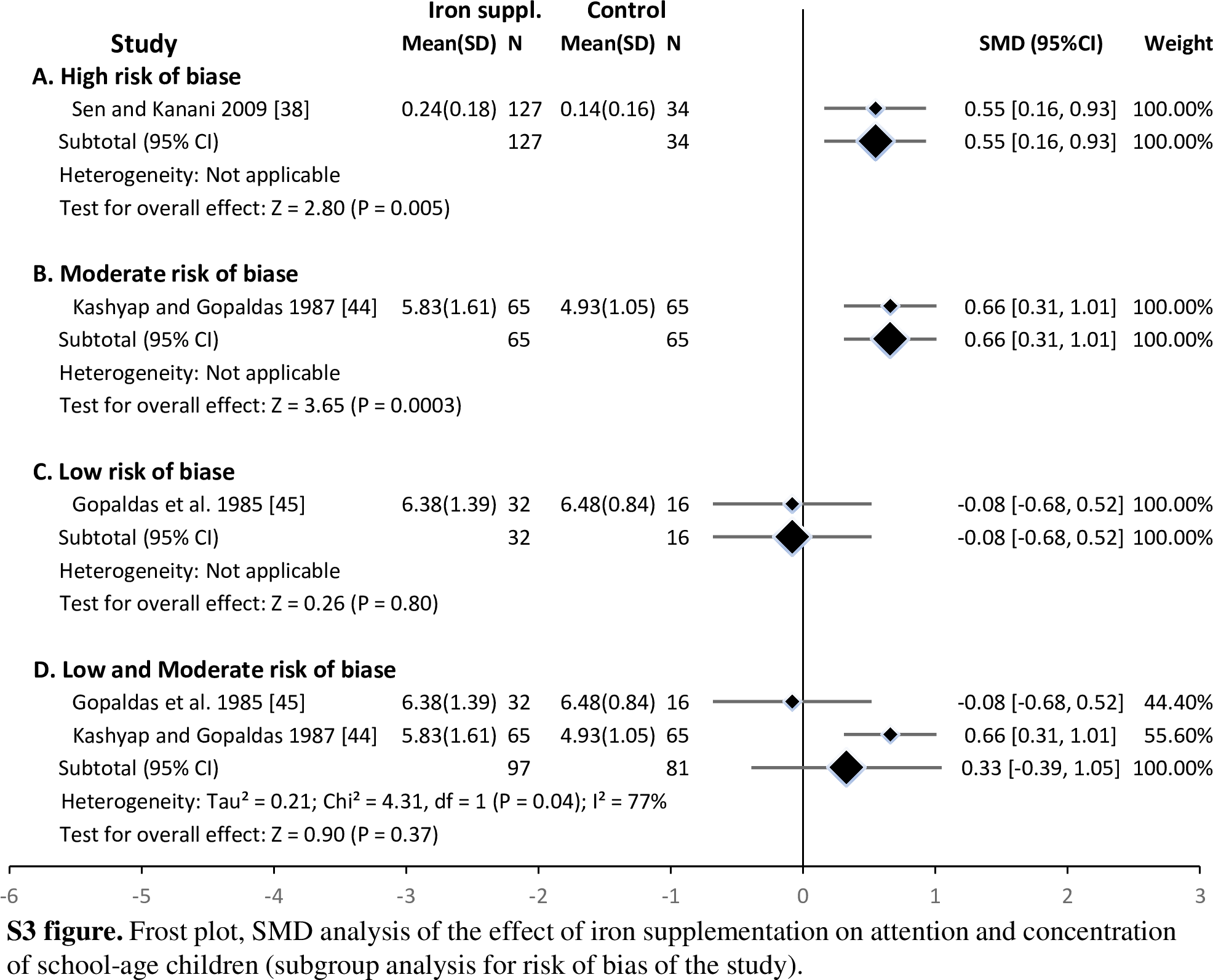

Supplement: S3 Fig — (TIF) [file pone.0287703.s009.tif]

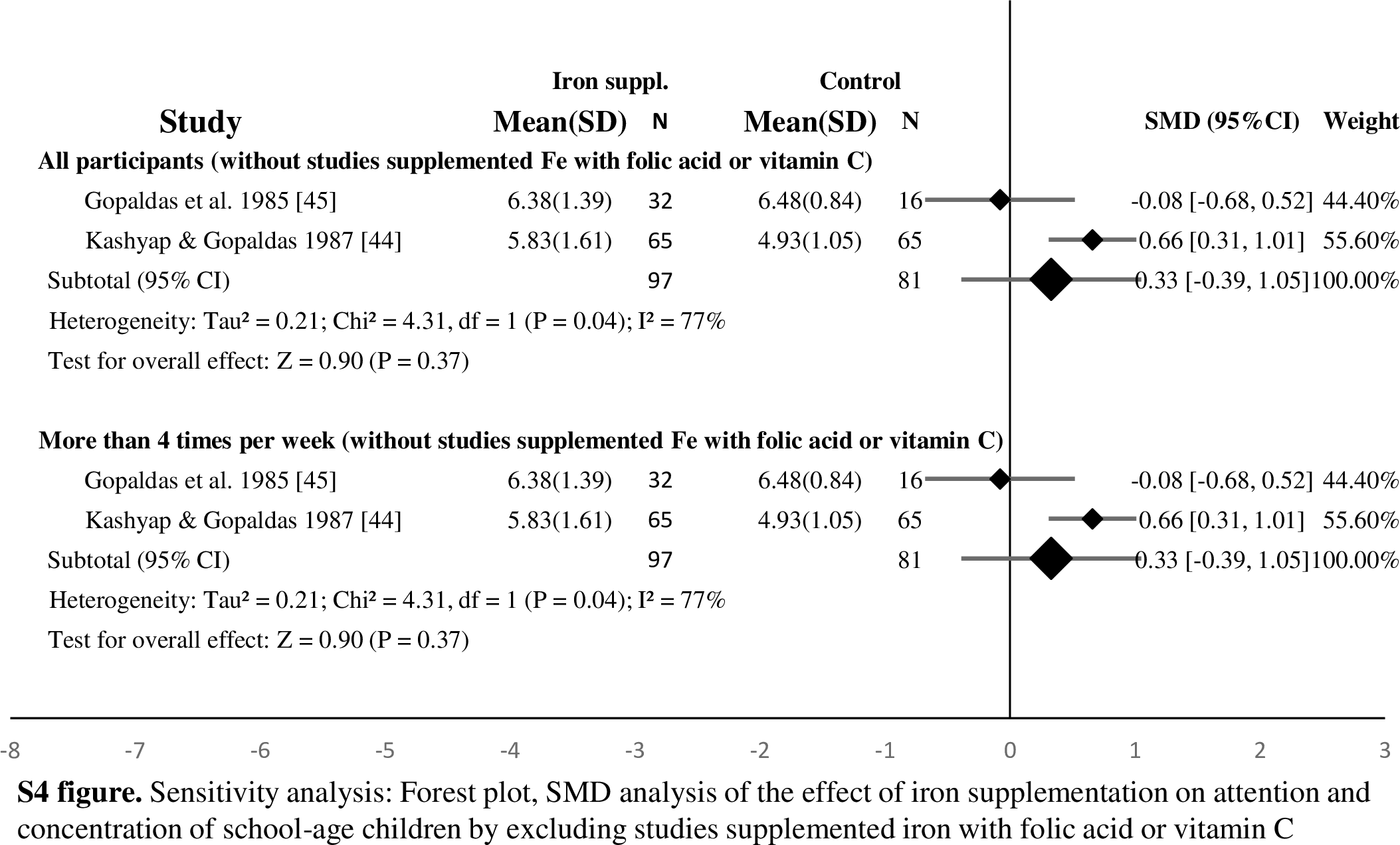

Supplement: S4 Fig — (TIF) [file pone.0287703.s010.tif]

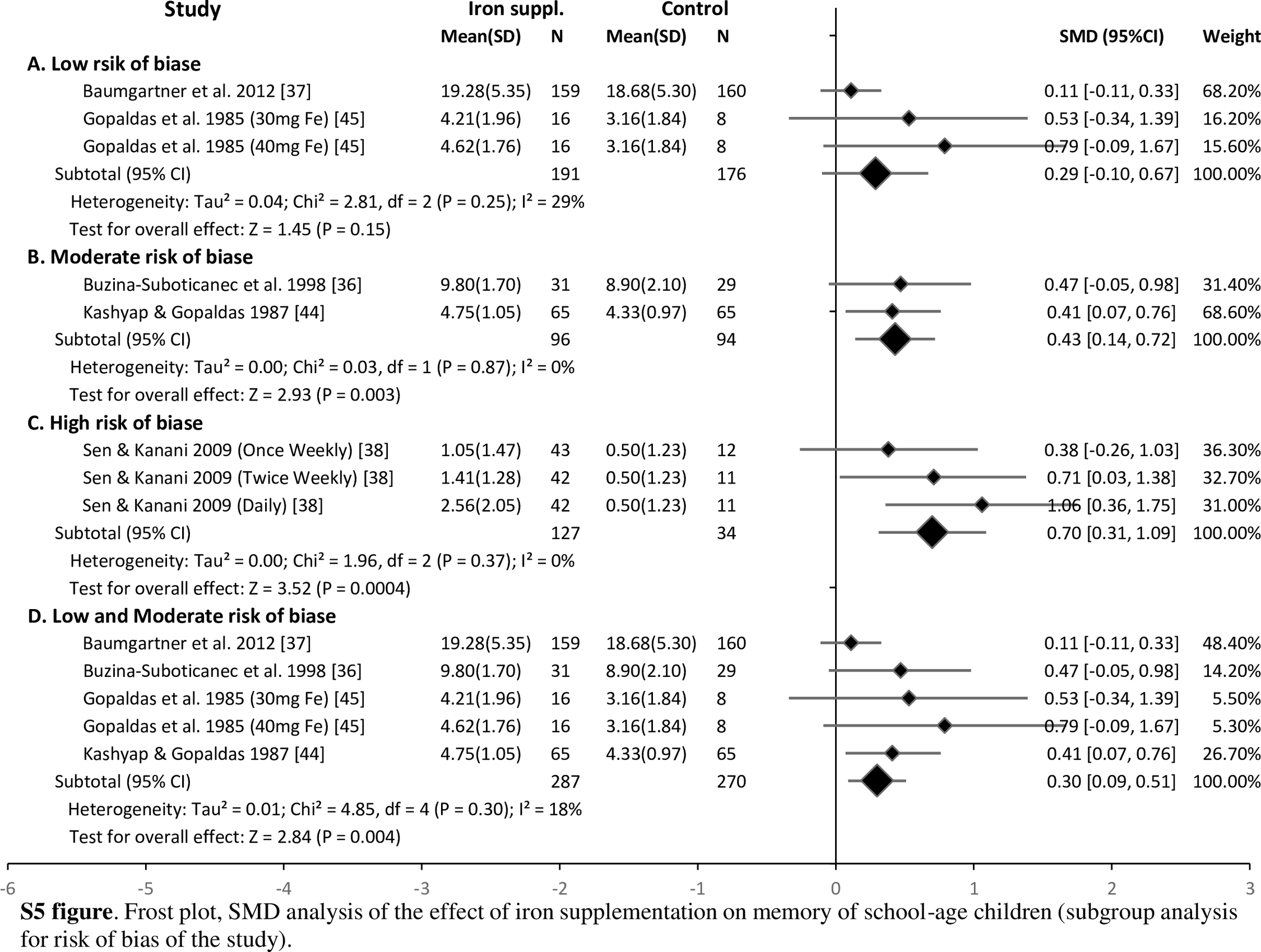

Supplement: S5 Fig — (TIF) [file pone.0287703.s011.tif]

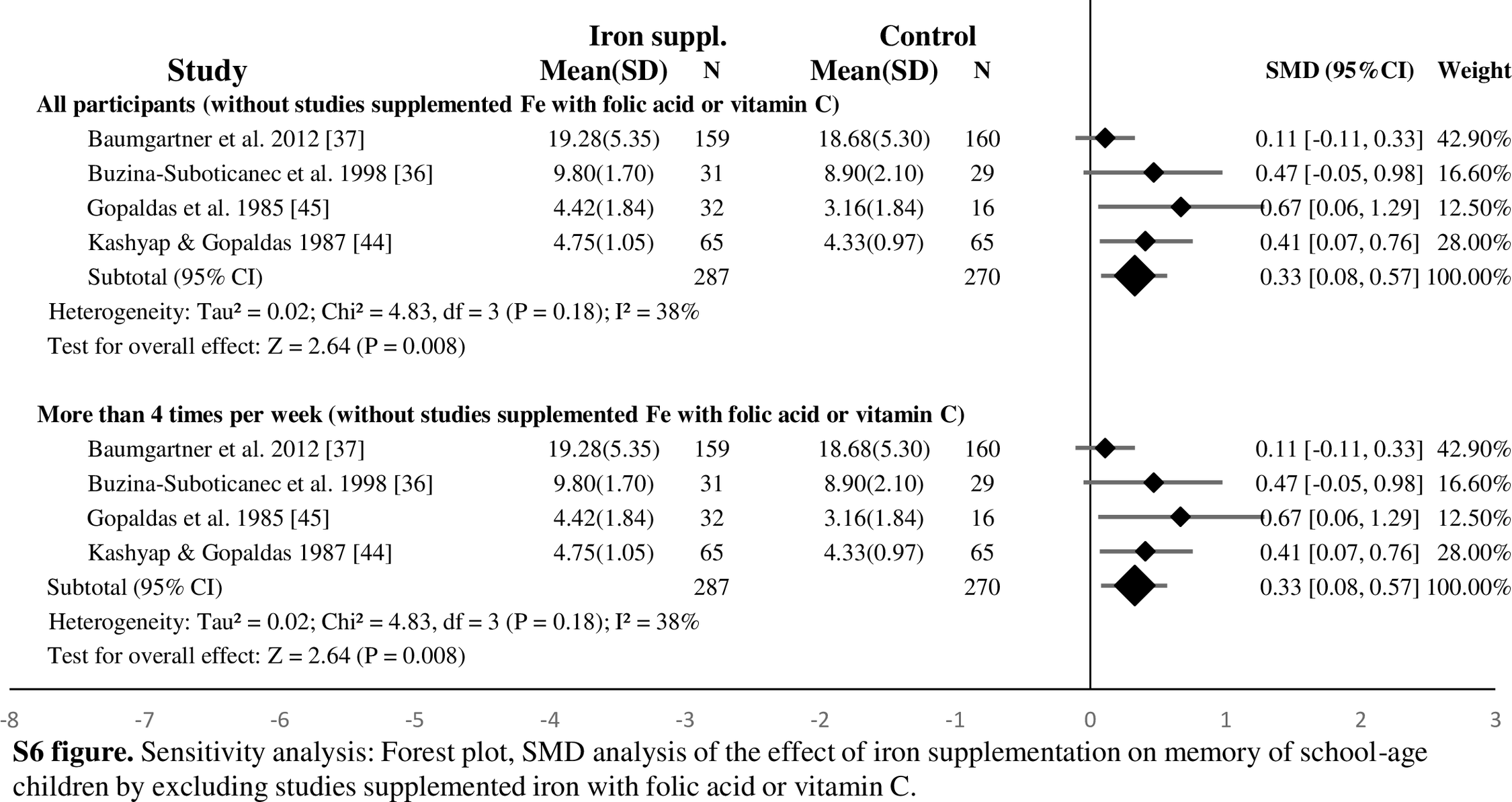

Supplement: S6 Fig — (TIF) [file pone.0287703.s012.tif]

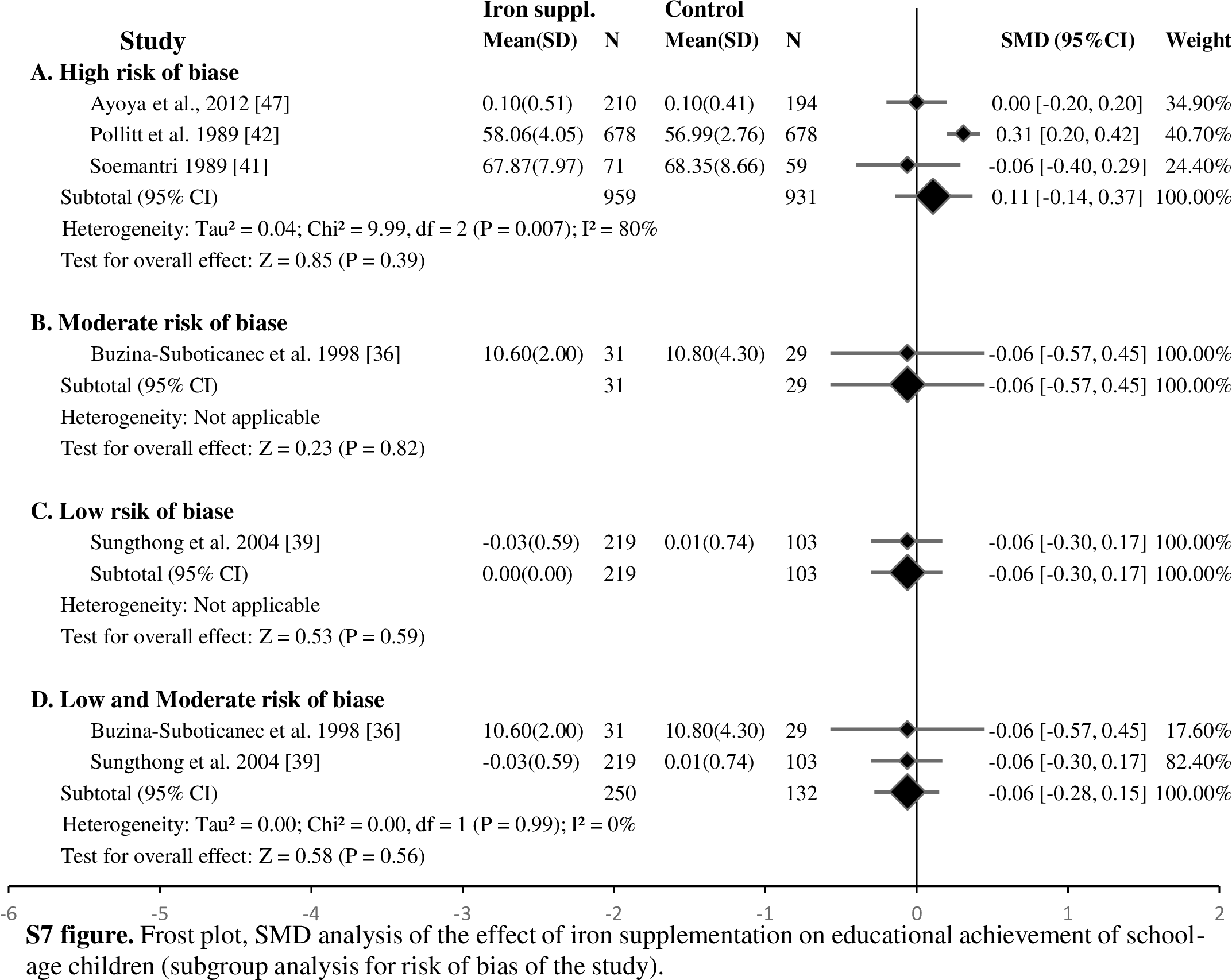

Supplement: S7 Fig — (TIF) [file pone.0287703.s013.tif]

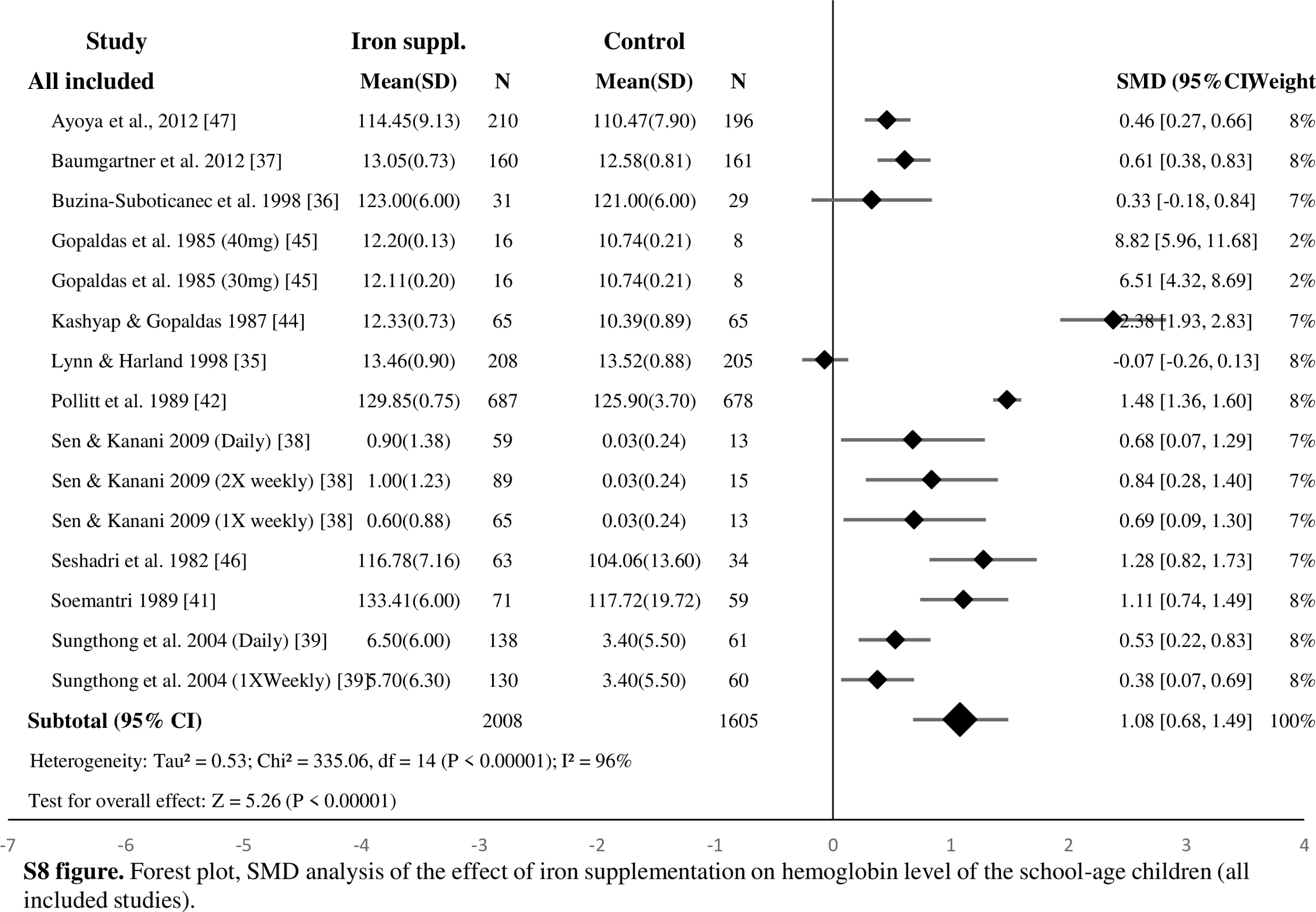

Supplement: S8 Fig — (TIF) [file pone.0287703.s014.tif]

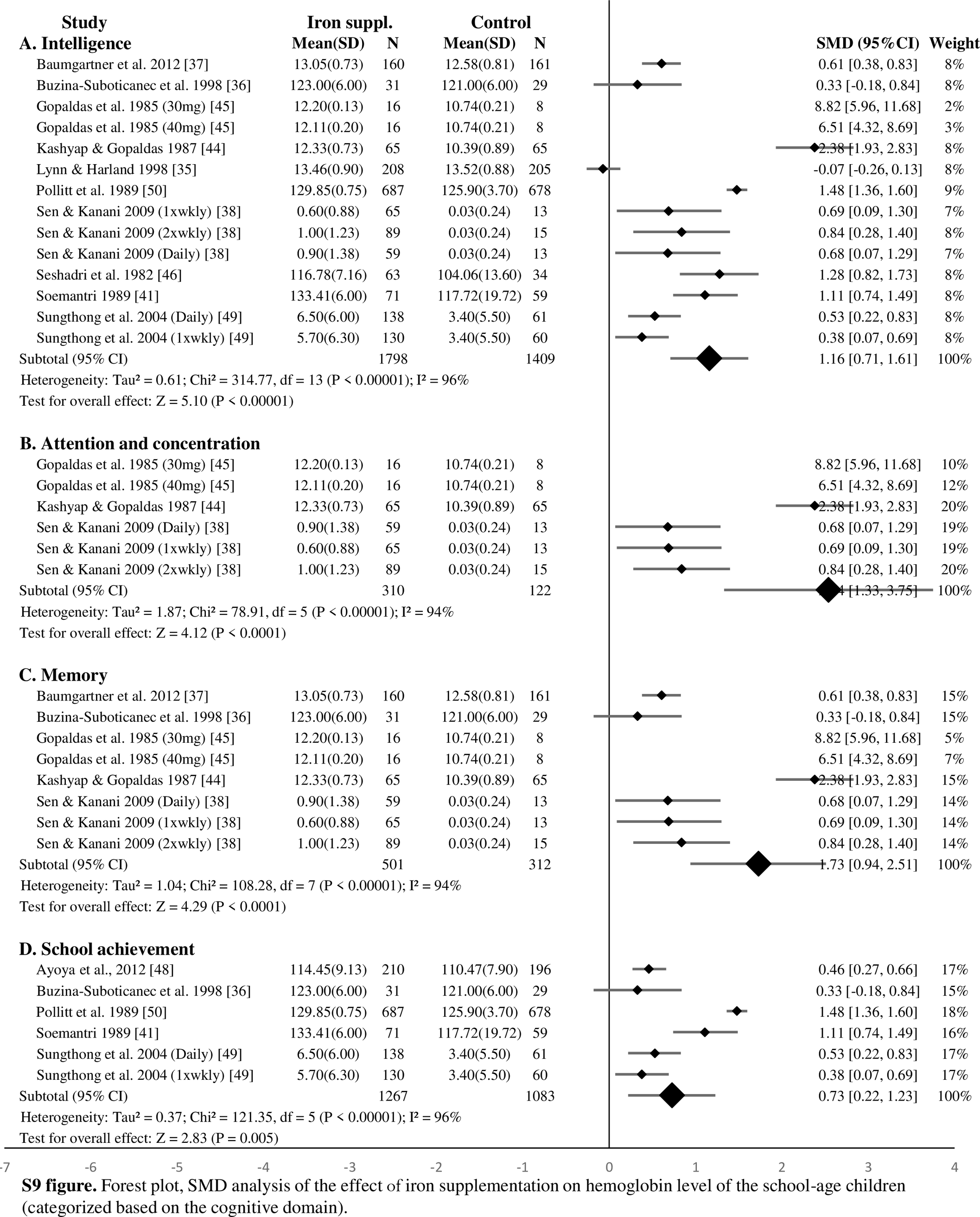

Supplement: S9 Fig — (TIF) [file pone.0287703.s015.tif]

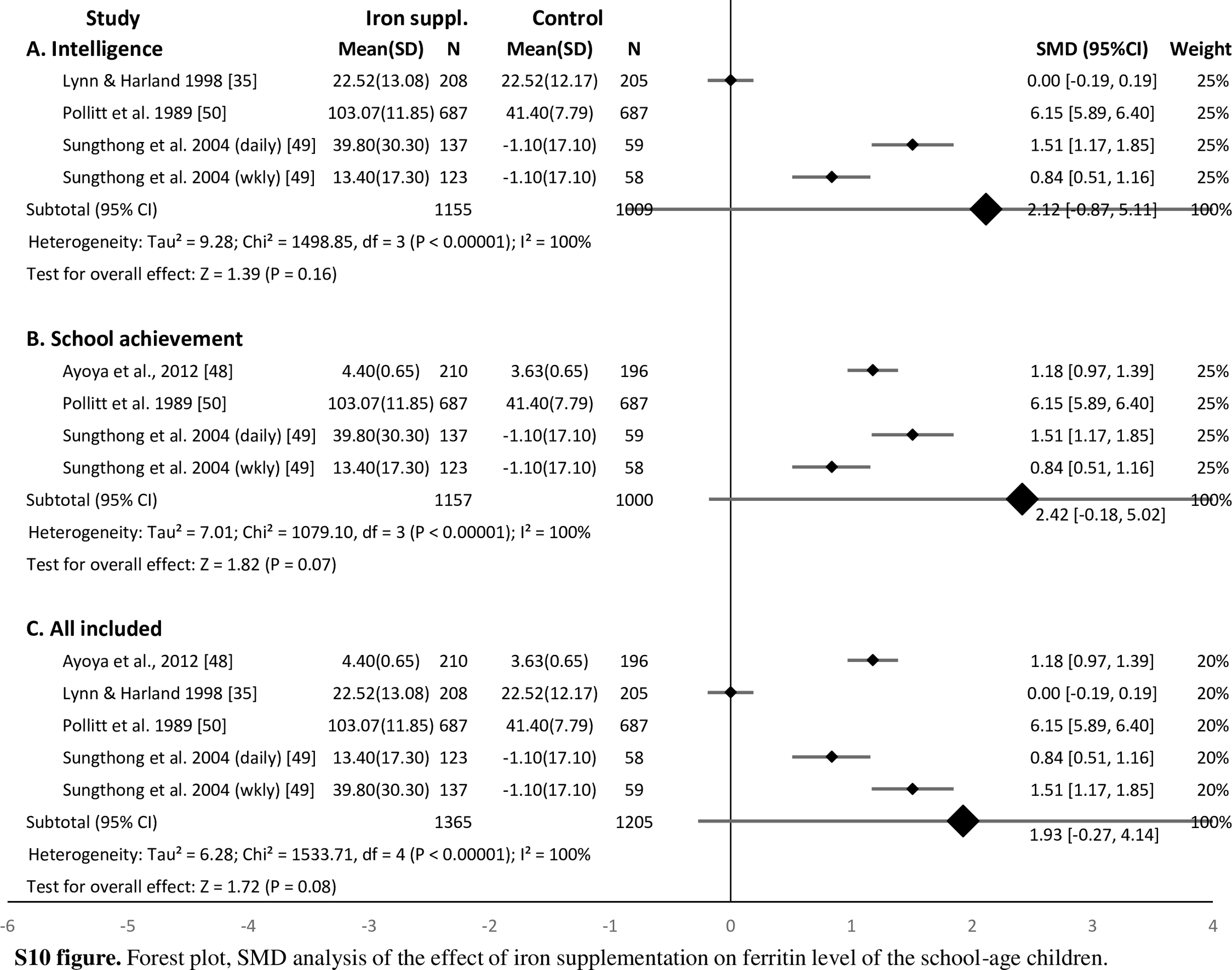

Supplement: S10 Fig — (TIF) [file pone.0287703.s016.tif]

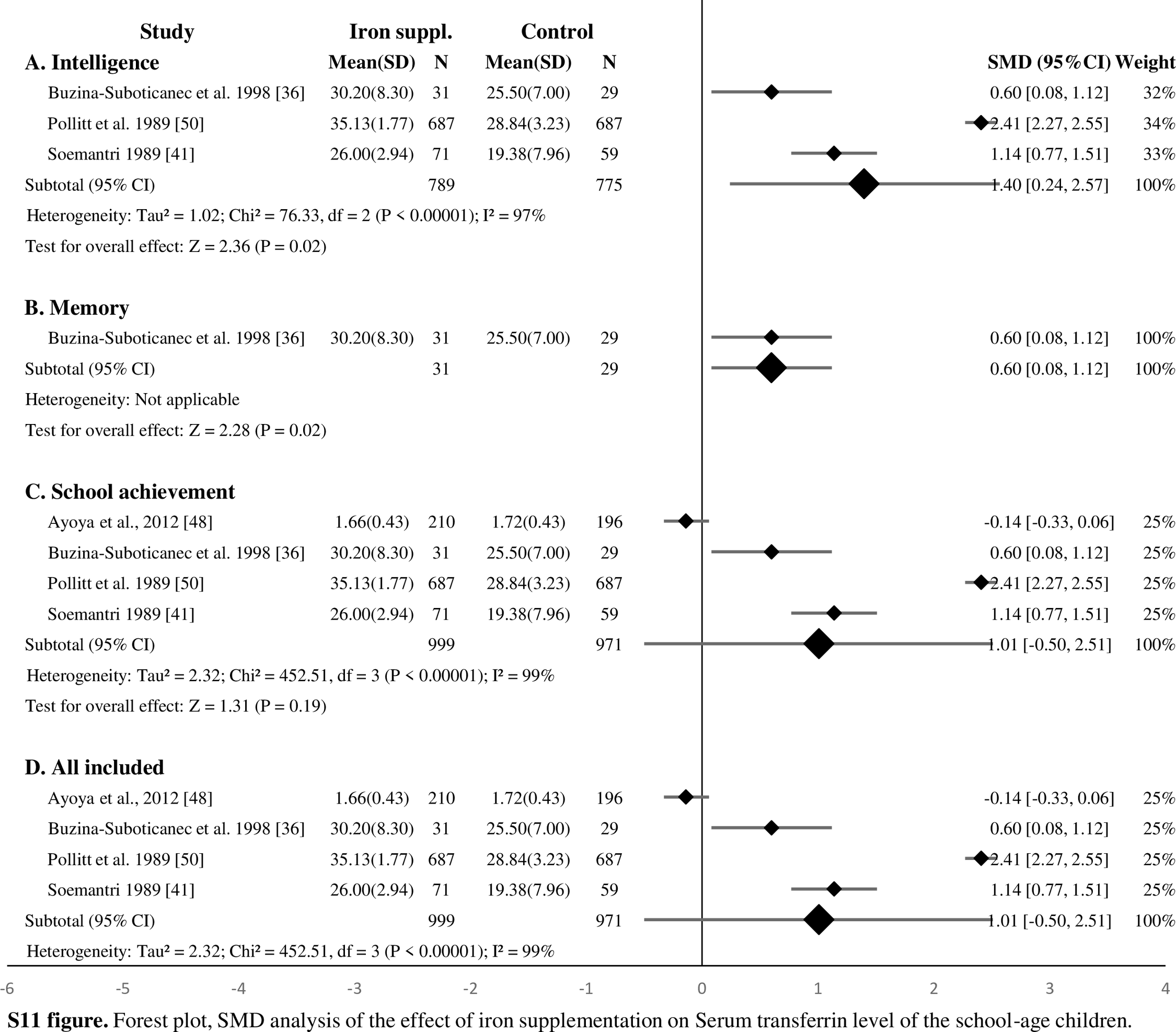

Supplement: S11 Fig — (TIF) [file pone.0287703.s017.tif]
