# Supplementary material for: Effects of iron supplementation on cognitive development in school-age children: Systematic review and meta-analysis
Source: PLoS One. 2023 Jun 27;18(6):e0287703. doi: 10.1371/journal.pone.0287703 (PMC10298800; doi:10.1371/journal.pone.0287703)
Supplement: Attachment — Submitted filename: Descision of the reviewer 23-12-2022.docx [file pone.0287703.s018.docx]

Thank you for referring the review on PLOS ONE manuscript “**Effects of preventive iron supplementation on cognitive development in school-age children: Systematic Review and Meta-analysis”** Manuscript Number: PONE-D-22-28873

The current manuscript is a systemic review and meta-analysis. This type of studies is at the top level of the evidence pyramid. It helps to settle controversies from conflicting studies and to determine whether new studies are needed for a given topic.

The topic of the study is very important as understanding the effects of iron supplementation on cognition among school age children is essential for the proper design of targeted strategies.

However, some aspects of the manuscript should be addressed:

**Title:**

“Effects of preventive iron supplementation on cognitive development in school-age children: Systematic Review and Meta-analysis”

I don’t understand what did the word (preventive) refer to? Did the authors intend to discuss iron supplementation to prevent iron deficiency anemia in school age children? I don’t think so, as the selected articles included anemic and non-anemic children.

**Introduction**

References in this section are not updated, most of them had been published one to two decades before.

This section lacks a paragraph illustrating the impact of iron deficiency status or iron deficiency anemia on cognitive development of school age children and consequently on their professional future.

Authors did not clarify the controversy between previous studies as regard the benefits and hazards of iron supplementation for this age group.

**Objective**

Is clear and well defined except for the word (preventive).

**Methods**

This section was written in a good technical standard and described in sufficient details. However, the part of statistical analysis was missed.

Methods of statistical analysis must be elaborated.

Readers must be prepared to understand the Forest plot figures which represented most of results. Values and significance of heterogeneity must be explained. If sensitivity analysis was applied or not.

Repeated sentences were detected in page 5 at lines 94-96.

As regards the definition of outcomes at page 5, I think no need for adding the biochemical outcomes as Hb concentration, serum ferritin level and transferrin saturation. These outcomes were not mentioned in the aims of the study and they will increase the diversity of results. Meanwhile, other outcomes as safety and adherence to the supplementation are closely related to the main outcomes.

**Results**

I was surprised that only one study out of the included 13 studies was performed in a low-income country, while anemia & iron deficiency are prevalent in these countries.

Comments on results were much confusing. These comments need rearrangement to rewrite the results of subgroups in a more structured manner. Authors can create a multilevel numbering to organize the comments on the main outcome domains and the underlying subgroups.

Results about safety outcome and adherence to the supplementation were missed.

**Discussion**

In the first page of discussion (page 20), the authors tried lengthily to prove that the studies included in the current review were also included in previous reviews, which added nothing to discussion.

The authors did not discuss explanation or hypothesis for the study findings. For example, the authors have to explain the underlying causes of non-improved school achievement in the studies included in this review, or non-improved cognitive functions in non-anemic participants, etc..

I think the authors need to revise the discussion to enrich it with causes of contradict or findings clarify the language, and adding updated references.

**Strengths and weaknesses**

These points were well documented.

**Conclusions**

Conclusions are presented in an organized and suitable manner.

**Decision: Minor revision**

**To the editor:**

A similar review has been published lately:

Chen, Z.; Yang, H.; Wang, D.; Sudfeld, C.R.; Zhao, A.; Xin, Y.; Chen, J.C.; Fawzi, W.W.; Xing, Y.; Li, Z. Effect of Oral Iron Supplementation on Cognitive Function among Children and Adolescents in Low- and Middle-Income Countries: A Systematic Review and Meta-Analysis. Nutrients 2022, 14, 5332. https://doi.org/10.3390/ nu14245332
