# Supplementary material for: Effects of iron supplementation on cognitive development in school-age children: Systematic review and meta-analysis
Source: PLoS One. 2023 Jun 27;18(6):e0287703. doi: 10.1371/journal.pone.0287703 (PMC10298800; doi:10.1371/journal.pone.0287703)
Supplement: Attachment — Submitted filename: 20230131 Plos One.docx [file pone.0287703.s019.docx]

I think this is a good systematic review and meta-analysis about the benefits of iron supplementation for the cognitive development in children. It has been well understood that in the school-aged children, iron supplementation has positive effects for improving age-adjusted height among anemic children, risk of anemia and iron deficiency, and the cognitive scores, including intelligence quotient, attention, and concentration.

Gutema et al has done a thorough work to apply their search strategy resulted in 6559 articles in the first phase and 1070 in the second phase. After which they screened again carefully and resulted in 13 articles included for the qualitative synthesis. We note that in 2022 there was a systematic review and meta-analysis published with the title of “Effect of Oral Iron Supplementation on Cognitive Function among Children and Adolescents in Low- and Middle-Income Countries: A Systematic Review and Meta-Analysis” which focused on studies in LMICs and in the age of children (and adolescents). Therefore, we acknowledge that Gutema et al implemented a different search strategy and inclusion criteria.

With the current title of “Effects of Preventive Iron Supplementation on Cognitive Development in School-Aged Children: Systematic Review and Meta-Analysis”, there are a few issues we would like to share:

1. The term ‘preventive’ used in the title does not seem to be suitable with the search strategy, analysis, and discussion. Page 26, Discussion section, line 389-390 also elaborated the aim of the review. Majority of the articles reviewed by Gutema et al recommended on the effects of iron in increasing the cognitive development parameters over 2 to 12 months supplementation. Therefore, we believe omitting the ‘preventive’ term would be more suitable.
2. The term ‘school-aged children’ does not match with the strategy applied by Gutema et al, because for example Gopaldas et al (1985 and 1987) included children aged 8-15 years, and Lynn and Harland (1998) included children aged 12-16 years.

We also would like to give input on some details below:

Page 8, line 39–40

Two keywords in the abstract: ‘iron supplementation’ and ‘school-aged children’ are not listed in the MeSH. We advise to choose the keywords based on the MeSH to increase the paper’s readability.

Page 10, line 81–82

“Studies conducted on children under five years or over 12 years of age were excluded.” However, Gopaldas et al (1985) and Kashyap and Gopaldas (1987) included children aged 8-15 years, Lynn and Harland (1998) included children aged 12-16 years, Sen and Kanani (2009) included children aged 9-13 years, Sungthong et al (2004) included children aged 6-13 years. Therefore, Gutema et al included studies in children aged more than 12 years old.

Page 10, line 82

The exclusion criteria applied by Gutema et al did not include other factors leading to bias, such as chronic morbidities and developmental disability. Whereas in Figure 1, there is an exclusion of 1 study due to Lead exposure. For this reason, we believe Gutema et al needs to add the exclusion criteria.

Page 10, line 87–89

“Observational study designs including case-report, case-series, ecological, cross-sectional, case-control, and cohort, quasi-experimental design, and reviews were excluded.”

This exclusion criteria is not needed because these other study designs are not included in the first place, as Gutema et al mentioned in the early paragraph that they only included RCTs.

Page 10 line 84-87

“Studies with multiple micronutrients are not included.” However, in page 14 line 178-180, some studies in combination with other supplementations (folic acid, albendazole, and ascorbic acid) were included in the review. While the studies involving EPA/DHA and multiple micronutrients were assessed properly by Gutema et al in separated groups.

Page 11 line 94–96

“The additional outcomes included anemia (hemoglobin concentrations below 115.0 g/l), hemoglobin concentrations (g/l), iron deficiency (measured using indicators of iron status including ferritin <15 μg/L and transferrin saturation <15%)”. We recommend the authors to mention the references for these cut off.

Page 11, line 106

“Screening in both phases was carried out by two reviewers (BTG, ND, MB, and GE), who independently included relevant articles and the above mentioned eligibility criteria. Disagreements were resolved by a third reviewer and consensus-based discussions. Screening of the updated records was carried out by one reviewer (BTG).”

We would like to confirm about the phases mentioned by the authors: Is the April search considered as the first phase, and the October search as the second phase? If they are, the authors need to inform about the reason for performing the second phase screening only by one person, while they stated that both phases involved two reviewers.

Page 19 line 220-221

“… daily 2 mg elemental iron”. This needs to be corrected to be 2 mg/kg, as is stated in the original paper, that is Soemantri (1989).

Table 2

Risk of bias for Sungthong (2004) indicated the paper has Low risk of bias in all domains (D1-D5). Figure 2 (Forest plot) suggested that iron daily and weekly were disadvantageous for cognitive development compared to placebo. We would suggest the authors to provide some possible explanations about this result, because it does not make sense for iron supplementations to have a more inferior outcome than ‘not giving iron supplementation’ (placebo). Gutema et al need to share their analysis about some factors in the study that could result in such unexpected outcome.

Table 2

The table indicated that Pollitt has a high overall risk of bias. Moreover, Figure 3 section D (iron deficient participants) displayed patients receiving iron supplementation has a lower SMD compared with the patients receiving placebo. We recommend Gutema et al to share their analysis about this result.

Figure 1

‘memroy’ is a typo, which is supposed to be memory.

Figure 3C

The figure showed that Pollit has only 100 patients with anemia and 1210 patients without anemia (in total is 1310 patients), whereas Table 1 mentioned that Pollit has 1775 subjects. Would it be possible there are significant numbers of missing data from Pollit?

Result section

Gutema et al occasionally mentioned the location name to refer to some particular studies, such as Kalibawang (when referring to Soemantri studies) and Vadodara (when referring to Sen and Kanani). I would suggest to mention the name of first author and year, i.e Soemantri (1985) than mentioning the place (Kalibawang).

Page 26, Discussion section

Gutema et al restated again about the inclusion and exclusion methods which we consider is unnecessary because these have been elaborated in the Results section.

Page 27 Line 419-420

Gutema et al need to mention ‘Figure 3’ as a reference for the description about prominent changes in the anemic groups.

Page 27 Discussion

For every aspect being compared, we believe the authors need to analyse about the reasons behind the results. The studies reviewed have different conclusions, which could be affected by the differences in population characteristics and dosages given. One example for this is the study by Sungthong (2004) which had significantly different outcomes compared with other studies, that is the placebo group had better outcomes than the iron-supplemented group.

Page 28 Strengths and Weaknesses of the Systematic Review and Meta-Analysis

From our perspectives, three limitations could be added, as follow:

1) Inability to conclude a specific dosage which would result in better cognitive development outcomes.

2) The observation period was relatively short: 2-3 months (in 5 studies), 4 months (in 5 studies), and 8-12 months (in 3 studies). The observation period in majority of studies could be too short for assessing a cognitive development.

3) The role of other micronutrients in some studies, that might affect the results.

Page 35, Figure 1

Records identified through database searching: 9041

Records after duplicates removed: 7669

Records screened: 6774

The authors need to provide information about what was done to reduce the number of articles from 7669 to 6774.
