# Supplementary material for: Effects of iron supplementation on cognitive development in school-age children: Systematic review and meta-analysis
Source: PLoS One. 2023 Jun 27;18(6):e0287703. doi: 10.1371/journal.pone.0287703 (PMC10298800; doi:10.1371/journal.pone.0287703)
Supplement: Attachment — Submitted filename: Reviewers decision after resubmission.docx [file pone.0287703.s021.docx]

Thank you for giving me the opportunity to revise the re-submitted manuscript PONE-D-22-28873R1 under the title: “**Effects of iron supplementation on cognitive development in school-age children: Systematic review and Meta-analysis”**

Thanks for the authors for their thorough revision of the manuscript and their earnest response to most of reviewers’ comments. I enjoyed revising this manuscript, and believe that it is very promising.

- The authors corrected the title of the manuscript as most of reviewers have asked
- They have updated the section of introduction by adding new references (from 2014-2022)
- They have illustrated briefly the impact of iron deficiency on cognitive development.
- They have added a statement regarding the safety of iron supplementation, but they didn’t illustrate the possible hazards of iron supplementation for this age group. Daily iron supplementation can cause gastrointestinal symptoms as upset stomach, constipation, nausea, abdominal pain, vomiting, and diarrhea. High doses of iron might also cause more serious effects, including inflammation of the stomach lining and ulcers. Therefore, it is necessary to pay attention to the suitable formula and the appropriate effective dose to avoid serious side effects.
- The objective has been rephrased.
- I think the added information concerned with statistical analysis were not sufficient.
- Adding eligibility criteria has enriched the methodology section. However, inclusion of studies provided folic acid or vitamin C together with iron may affect the precision of analysis due to the known effect of folic acid on cognition and the enhancement of iron absorption induced by vitamin C.
- Though it was better to control the duration, frequency, and dose of iron supplementation to avoid the risk of bias. Practically, it is difficult to uniform the design of the available intervention trials.
- Authors have restructured results on the base of subgroup analysis, and have added the results of the previously missed parts about safety and adherence to the supplementation.
- They have modified the section of discussion to omit unnecessary paragraphs and add needed ones. However, they didn’t provide explanations for the unexpected outcomes. Some findings are still ambiguous (e.g., there was no effect of iron supplementation on the intelligence of children who were iron deficient at baseline. Iron-depleted children assigned to placebo, scored higher in RCPM test (IQ) at baseline compared to iron-depleted children who received the iron treatment [42]).
- I appreciate all authors efforts and consider this manuscript as an important addition to the field of child mental development.
