# Supplementary material for: Effects of iron supplementation on cognitive development in school-age children: Systematic review and meta-analysis
Source: PLoS One. 2023 Jun 27;18(6):e0287703. doi: 10.1371/journal.pone.0287703 (PMC10298800; doi:10.1371/journal.pone.0287703)
Supplement: Attachment — Submitted filename: Rebuttal Letter PONE-D-22-28873 R2.docx [file pone.0287703.s022.docx]

Ref.:

PONE-D-22-28873R1

Effects of iron supplementation on cognitive development in school-age children: Systematic review and Meta-analysis

PLOS ONE

Dear Dr. Gutema,

Thank you for submitting your manuscript to PLOS ONE. After careful consideration, we feel that it has merit but does not fully meet PLOS ONE’s publication criteria as it currently stands. Therefore, we invite you to submit a revised version of the manuscript that addresses the points raised during the review process.

Please submit your revised manuscript by Jul 14 2023 11:59PM. If you will need more time than this to complete your revisions, please reply to this message or contact the journal office at [plosone@plos.org](mailto:plosone@plos.org). When you're ready to submit your revision, log on to [https://www.editorialmanager.com/pone/](https://eur03.safelinks.protection.outlook.com/?url=https%3A%2F%2Fwww.editorialmanager.com%2Fpone%2F&data=05%7C01%7CSouheila.Abbeddou%40UGent.be%7C6d6e224b6da14f57abe008db62b41fb9%7Cd7811cdeecef496c8f91a1786241b99c%7C1%7C0%7C638212298899115472%7CUnknown%7CTWFpbGZsb3d8eyJWIjoiMC4wLjAwMDAiLCJQIjoiV2luMzIiLCJBTiI6Ik1haWwiLCJXVCI6Mn0%3D%7C3000%7C%7C%7C&sdata=0ZbJcMvXLthpHW%2Bfi6l7I%2Bh3VnVjlnrssJvTZ%2FCeBKw%3D&reserved=0) and select the 'Submissions Needing Revision' folder to locate your manuscript file.

Please include the following items when submitting your revised manuscript:

- A rebuttal letter that responds to each point raised by the academic editor and reviewer(s). You should upload this letter as a separate file labeled 'Response to Reviewers'.
- A marked-up copy of your manuscript that highlights changes made to the original version. You should upload this as a separate file labeled 'Revised Manuscript with Track Changes'.
- An unmarked version of your revised paper without tracked changes. You should upload this as a separate file labeled 'Manuscript'.

If you would like to make changes to your financial disclosure, please include your updated statement in your cover letter. Guidelines for resubmitting your figure files are available below the reviewer comments at the end of this letter.

If applicable, we recommend that you deposit your laboratory protocols in protocols.io to enhance the reproducibility of your results. Protocols.io assigns your protocol its own identifier (DOI) so that it can be cited independently in the future. For instructions see: [https://journals.plos.org/plosone/s/submission-guidelines#loc-laboratory-protocols](https://eur03.safelinks.protection.outlook.com/?url=https%3A%2F%2Fjournals.plos.org%2Fplosone%2Fs%2Fsubmission-guidelines%23loc-laboratory-protocols&data=05%7C01%7CSouheila.Abbeddou%40UGent.be%7C6d6e224b6da14f57abe008db62b41fb9%7Cd7811cdeecef496c8f91a1786241b99c%7C1%7C0%7C638212298899115472%7CUnknown%7CTWFpbGZsb3d8eyJWIjoiMC4wLjAwMDAiLCJQIjoiV2luMzIiLCJBTiI6Ik1haWwiLCJXVCI6Mn0%3D%7C3000%7C%7C%7C&sdata=XZ1J81Jp7vlMufqMDMulmX%2B%2FvCPX4UAnKr2UGBbAUTc%3D&reserved=0). Additionally, PLOS ONE offers an option for publishing peer-reviewed Lab Protocol articles, which describe protocols hosted on protocols.io. Read more information on sharing protocols at [https://plos.org/protocols?utm_medium=editorial-email&utm_source=authorletters&utm_campaign=protocols](https://eur03.safelinks.protection.outlook.com/?url=https%3A%2F%2Fplos.org%2Fprotocols%3Futm_medium%3Deditorial-email%26utm_source%3Dauthorletters%26utm_campaign%3Dprotocols&data=05%7C01%7CSouheila.Abbeddou%40UGent.be%7C6d6e224b6da14f57abe008db62b41fb9%7Cd7811cdeecef496c8f91a1786241b99c%7C1%7C0%7C638212298899271693%7CUnknown%7CTWFpbGZsb3d8eyJWIjoiMC4wLjAwMDAiLCJQIjoiV2luMzIiLCJBTiI6Ik1haWwiLCJXVCI6Mn0%3D%7C3000%7C%7C%7C&sdata=%2BNQzrKBd5Bij3js0c2qNuLJwKuqL3Y3zifhiv9Pg8o8%3D&reserved=0).

We look forward to receiving your revised manuscript.

Kind regards,

Ammal Mokhtar Metwally, Ph.D (MD)

Academic Editor

PLOS ONE

Dear Dr. Ammal Mokhtar Metwally,

Thank you for your reply sent on May 31, 2023.

Enclosed we are submitting the revised manuscript PONE-D-22-28873R1, entitled “Effects of iron supplementation on cognitive development in school-age children: Systematic review and Meta-analysis”

By Befikadu Tariku Gutema, Muluken Bekele Sorrie, Nega Degefa Megersa, Gesila Endashaw Yesera, Yordanos Gizachew Yeshitila, Nele S. Pauwels, Stefaan De Henauw, and Souheila Abbeddou.

We thank the reviewers for their constructive comments, which helped us to improve our paper. Our responses addressing the concerns of the reviewers are provided below, and all changes in the manuscript are marked with track changes. We hope that the revisions meet your expectations and that the manuscript will now be acceptable for publication.

Sincerely,

Befikadu Tariku Gutema

# Reviewer #1

Reviewer #1: Thank you for giving me the opportunity to revise the re-submitted manuscript PONE-D-22-28873R1 under the title: “Effects of iron supplementation on cognitive development in school-age children: Systematic review and Meta-analysis”

Thanks for the authors for their thorough revision of the manuscript and their earnest response to most of reviewers’ comments. I enjoyed revising this manuscript, and believe that it is very promising.

Thank you for your very positive feedback. We will address the remaining points.

• The authors corrected the title of the manuscript as most of reviewers have asked

Indeed.

• They have updated the section of introduction by adding new references (from 2014-2022)

Yes, we thank the reviewer as well from brining this up.

• They have illustrated briefly the impact of iron deficiency on cognitive development.

Yes, that you.

• They have added a statement regarding the safety of iron supplementation, but they didn’t illustrate the possible hazards of iron supplementation for this age group. Daily iron supplementation can cause gastrointestinal symptoms as upset stomach, constipation, nausea, abdominal pain, vomiting, and diarrhea. High doses of iron might also cause more serious effects, including inflammation of the stomach lining and ulcers. Therefore, it is necessary to pay attention to the suitable formula and the appropriate effective dose to avoid serious side effects.

Response: We agree that this part should be added to the discussion. Now added under the paragraph Lines 515-526 which reads:

Concerns regarding possible side effects

Iron supplementation can cause gastrointestinal side effects including loose stool/diarrhea, hard stool/constipation, abdominal pain, nausea, change in stool color, reflux/heartburn and headache [67]. Low doses of iron supplementation are reportedly not associated with an increased risk of infectious illness in children, except for a slight increase in the risk of developing diarrhea. Iron supplementation may increase the risk of malaria [reference 63], and the WHO recommends monitoring fever in preschool and school children receiving intermittent iron supplementation in malaria settings [22]. Despite the serious risk that is evidenced with iron supplementation, side effects were reported in two studies only [51, 52], while the increased risk of malaria infection was reported in one study only [48] and three studies indicated that the setting was malaria-free [42, 49, 51]. It is important that morbidity and potential side effects are assessed in studies and programs that supplement iron to children.

• The objective has been rephrased.

Indeed, we rephrased it based on the previous revision.

• I think the added information concerned with statistical analysis were not sufficient.

Response: Now, on the tope of sensitivity analysis for the studies for trials with multiple form of supplementation of iron (frequency of administration or dose difference) we included to check iron supplementations combined with other micronutrients, and to check the effect of each study on the overall effect of the intervention by removing one trial at a time.

• Adding eligibility criteria has enriched the methodology section. However, inclusion of studies provided folic acid or vitamin C together with iron may affect the precision of analysis due to the known effect of folic acid on cognition and the enhancement of iron absorption induced by vitamin C.

Response: These studies were included because of the form that is commonly distributed of iron with folic acid. But we agree that this could affect the analysis. We have carried out a sensitivity analysis and included the finding in the document.

• Though it was better to control the duration, frequency, and dose of iron supplementation to avoid the risk of bias. Practically, it is difficult to uniform the design of the available intervention trials.

Response: Definitely, with the diverse design of the studies, it is almost impossible to uniform the findings. However, we tried in the subgroup analyses to provide at least an insight.

• Authors have restructured results on the base of subgroup analysis, and have added the results of the previously missed parts about safety and adherence to the supplementation.

Thanks.

• They have modified the section of discussion to omit unnecessary paragraphs and add needed ones. However, they didn’t provide explanations for the unexpected outcomes. Some findings are still ambiguous (e.g., there was no effect of iron supplementation on the intelligence of children who were iron deficient at baseline. Iron-depleted children assigned to placebo, scored higher in RCPM test (IQ) at baseline compared to iron-depleted children who received the iron treatment [42]).

Response: The controversial results were driven by the study of Pollitt et al. (1989), where indeed baseline data were different between placebo and intervention groups. After conducting the same analysis without this study, the result showed significant improvement in cognition among iron deficient children in intelligence. We included the following in the result part. Lines 294-297 which reads: “The sensitivity analysis showed that iron supplementation significantly improved the intelligence of iron deficient children (SMD 0.52, 95%CI: 0.16, 0.87, P= 0.005, n=128; test for heterogeneity I2=0%, P=0.77) after dropping the study of Pollitt et al. (1989) [42].”

• I appreciate all authors efforts and consider this manuscript as an important addition to the field of child mental development.

We thank the reviewer and we hope that the additional revisions meet your expectations.

# Reviewer #2:

Reviewer #2: I greatly appreciate the authors' effort to improve the manuscript. The authors have done a nice job of revising this manuscript and addressing the reviewers' questions/issues raised in the initial review. The revised manuscript is more logical; I am able to understand the methodology, study flow, and results. I recommend that the revised paper be accepted with very minor revisions. My comments below are related to the Conclusion presented in the paper.

We thank the reviewer for their very positive feedback and for their comments that helped us improve the manuscript.

I recommend the authors delete this sentence because it is irrelevant to the results, discussion, and study aim. Furthermore, the statement could be misunderstood as suggesting that a short duration of iron supplementation can be beneficial: "Targeting anemic children, and a more frequent supplementation for shorter duration showed promising results."

Response: The sentence has been dropped as recommended.

# Reviewer #3:

I have reviewed the revised version of the manuscript from Gutema et al. I sincerely want to say thank you for the major improvements in the writings, especially in correcting for the typographical errors and other certain incorrect use of words in a sentence. The comments from other reviewer have also been addressed. However, I suggest to put the full form of abbreviated words as they first appeared on the text (e.g. RoB hasn't been explained until the end of the manuscript, the full word for RCPM hasn't been written) before this manuscript will be published.

Response: Corrected. We added the abbreviation Under 'Methods and design in subtitle ‘Assessment of risk of bias’ part. The risk of bias (RoB)…. Regarding RCPM, the abbreviation was only at one point. The others were written in full. So, we replaced abbreviation by the full word.

The discussion sections is greatly improved and the authors have put great reasoning and comparations within their findings. I'm looking forward for this manuscript to be published as this study will provide important insights upon iron supplementation on cognitive development in children.

We thank you for the very positive evaluation.
